# Comment on: Quality of life after open *versus* laparoscopic distal pancreatectomy: long-term results from a randomized clinical trial

**DOI:** 10.1093/bjsopen/zrad070

**Published:** 2023-07-17

**Authors:** Sharon Barker, Agastya Patel, Thomas Satyadas

**Affiliations:** Department of Hepato-Pancreato-Biliary Surgery, Manchester Royal Inﬁrmary, Manchester, UK; Department of Hepato-Pancreato-Biliary Surgery, Manchester Royal Inﬁrmary, Manchester, UK; Department of General, Endocrine and Transplant Surgery, First Doctoral School of Medical University of Gdansk, Gdansk, Poland; Department of Hepato-Pancreato-Biliary Surgery, Manchester Royal Inﬁrmary, Manchester, UK

*Dear Editor*

We read with great interest the article by Johansen *et al*. assessing quality of life (QoL) of patients undergoing minimally invasive distal pancreatectomy (MIDP) and open distal pancreatectomy (ODP) in a randomized clinical trial setting^1^. It is encouraging to observe the increasing interest in patient-specific outcomes to inform clinical decision-making.

It is well known that multimodal surgical prehabilitation, including exercise, nutrition, smoking/alcohol cessation, and psychological counselling, improves postoperative outcomes as well as long-term QoL^2,3^. Based on the LAPOP trial protocol and the present study, we were unable to determine the prehabilitation programme the participants underwent leading up to surgery. Additionally, it would be interesting to assess whether there were differences in length of prehabilitation and adherence with individual components between the arms, as these are crucial factors affecting postoperative outcomes^4^.

A key result of the present study is demonstrating that patients within the MIDP arm had similar scores in several QoL domains at 2 years, compared with a matched Swedish general population. One of the important confounders in QoL analysis is socioeconomic factors, such as education level, income status etc. A recent Swedish study identified that lower socioeconomical and professional status is associated with poorer postoperative health-related QoL^5^. Hence, it is also important to assess whether the arms differed in terms of their socioeconomic status, and how this might influence the long-term QoL outcomes following distal pancreatectomy.

In conclusion, Johansen *et al*. provide seminal data in relation to long-term QoL following MIDP and ODP, which adds to the rationale of the already standard minimally invasive approach for distal pancreatectomy. However, further information on the aforementioned variables may provide a more complete picture of the issue at hand. Lastly, as the majority of patients had benign indications, there remains a need to research the impact of rehabilitation/supportive interventions on QoL following DP for oncological indications, where more extensive support may be required.

## Disclosure

The authors declare no conflict of interest.
